# Supplementary material for: Multicenter Experience with Good Manufacturing Practice Production of [11C]PiB for Amyloid Positron Emission Tomography Imaging
Source: Pharmaceuticals (Basel). 2024 Feb 7;17(2):217. doi: 10.3390/ph17020217 (PMC10892710; doi:10.3390/ph17020217)
Supplement: Supplementary file 1 [file pharmaceuticals-17-00217-s001.zip › pharmaceuticals-2822546-supplementary.pdf]

Multicenter experience with GMP production of [^{11}C]PiB for amyloid PET imaging

Anders B. A. Andersen[1], Szabolcs Lehel[2], Ebbe K. Grove[3], Niels Langkjær[4], Dan Fuglø[1], and Tri Hien Viet Huynh[1]

[1]*Department of Nuclear Medicine, Copenhagen University Hospital, Herlev and Gentofte, Denmark;* [2]*Department of Clinical Physiology, Nuclear Medicine and PET, Copenhagen University Hospital, Rigshospitalet, Denmark;* [3]*Department of Nuclear Medicine and PET Centre, Aarhus University Hospital, Aarhus, Denmark;* [4]*Department of Nuclear Medicin, Odense University Hospital, Denmark.*

For correspondence contact: Tri Hien Viet Huynh, Department of Nuclear Medicine, Copenhagen University Hospital, Herlev and Gentofte, Borgmester Ib Juuls Vej 31, DK-2730 Herlev, Denmark.

Email: THUY0011@regionh.dk

Contents

S1: Radiosynthesis of [^{11}C]PiB at AUH **S3**
- **Figure S1: Schematic overview of TRACERlab FX C Pro setup at AUH** **S6**
- **Figure S2: Overall [^{11}C]PiB synthesis flowchart at AUH** **S7**
- **Figure S3: TRACERlab FX C Pro setup for [^{11}C]PiB synthesis at AUH** **S8**
- **Figure S4: Semi-preparative HPLC traces for [^{11}C]PiB production at AUH** **S8**

S2: Radiosynthesis of [^{11}C]PiB at HUH **S9**
- **Figure S5: TracerMaker setup for [^{11}C]PiB synthesis at HUH** **S11**
- **Figure S6: Schematic overview of the TracerMaker setup at HUH** **S12**
- **Figure S7: Overall [^{11}C]PiB synthesis flowchart at HUH** **S13**
- **Figure S8: Radio chromatogram of [^{11}C]PiB product isolation at HUH** **S14**

S3: Radiosynthesis of [^{11}C]PiB at OUH **S15**
- **Figure S9: Schematic overview of TRACERlab FX C Pro setup at OUH** **S17**
- **Figure S10: Overall [^{11}C]PiB synthesis flowchart at OUH** **S18**

S4: Radiosynthesis of [^{11}C]PiB at RH **S19**
- **Figure S11: ScanSys setup for [^{11}C]PiB synthesis at RH** **S23**
- **Figure S12: TracerMaker setup for [^{11}C]PiB synthesis at RH** **S23**
- **Figure S13: Schematic overview of the ScanSys setup at RH** **S24**
- **Figure S14: Schematic overview of the TracerMaker setup at RH** **S25**
- **Figure S15: Semi-preparative HPLC traces at RH** **S26**

S5: Quality Control and Specifications for [^{11}C]PiB for all sites **S27**
- **Table S1: [^{11}C]PiB product specifications for all sites** **S28**
- **Table S2: Quality control equipment and parameters for all sites** **S29**

S6: Synthesis Module Columns - Details and Preparation **S30**
- **Table S3: Packing materials and dimensions of the columns at AUH** **S30**
- **Table S4: Packing materials and dimensions of the columns at OUH** **S31**
- **Table S5: Packing materials and dimensions of the TracerMaker at HUH/RH** **S32**
- **Table S6: Packing materials and dimensions of the ScanSys at RH** **S33**

S1: Radiosynthesis of [^{11}C]PiB at AUH

At AUH, [^{11}C]PiB is produced on a TRACERlab FX C Pro automated synthesis module by use of [^{11}C]CO_2 or [^{11}C]CH_4. However, [^{11}C]CO_2 affords higher yields and is preferably used. See Figure S1 for a schematic overview in the TRACERlab software, Figure S2 for a flowchart of the overall synthesis and quality control (QC), and Figure S2 for a picture of the TRACERlab FX C Pro setup. The [^{11}C]CO_2 is generated either by a GE PETtrace 600, a GE PETtrace 800, or an IBA Cyclone 18/18 cyclotron. Bombardment of 99.5% N_2 + 0.5% O_2 (Air Liquide Denmark A/S) for [^{11}C]CO_2 production or 95% N_2 + 5% H_2 (Air Liquide Denmark A/S) for [^{11}C]CH_4 production is performed with an average irradiation time and beam current of 44.5 min and 52.7 μA, respectively (Table 3, main text).

Preparation and cleaning of TRACERlab FX C Pro

Prior to each production, the TRACERlab FX C Pro synthesis module and the HPLC loop are cleaned and dried. For cleaning, the vials of the module are loaded as follows (see Figs. S1 and S3): Vial 1; EtOH, 70% (1 mL); Vial 2: EtOH, 70% (1 mL); Vial 3: EtOH, 70% (2 mL), Vial 4: EtOH, 70% (10 mL), Vial 5: EtOH, 70% (2 mL), Vial 6: EtOH, 70% (10 mL); and round bottomed flask (RBF): EtOH, 70% (10 mL). Upon module cleaning, the EtOH from Vial 1 is initially passed through the reactor, v8 and v7 to the waste collection bottle. Then EtOH from Vial 2 and 3 is passed through the reactor, v8 and v7 to waste. The vials are then dried by a flow of helium from v18. EtOH from Vial 4 and 6 is passed through v11 and send to the waste collection bottle through v12. Lastly, EtOH from vial 5 is passed through v11 and v12 to the product flask and further through v13 to waste.

The module is then loaded again as follows (Figure S1 and S3): Vial 4: sterile water (10 mL); Vial 6: sterile water (10 mL); and RBF: sterile water (10 mL). The sterile water from RBF is flushed to the waste collection bottle through v11 and v12. The sterile water from Vial 4 is then

passed through v11 and v12 to the product flask and further to waste through v13. The sterile water from Vial 6 is flushed to waste through v11 and v12. The reactor is heated to 100 °C and a stream of helium is passed through Vial 1 and 2 to the reactor with vacuum. Lastly, a flow of helium is sent through vial 3 for drying.

The TRACERlab FX C Pro synthesis module is then prepared in the following way for [^{11}C]PiB synthesis (Figure S1 and S3): a 5 mL stainless steel loop; a variable wavelength UV detector (Knauer): 254 nm; precursor: 6-OH-BTA-0 (2.0 ± 0.2 mg, 7.4-9.1 μmol) in acetone (300 μL); Vial 3: 1 mL sterile water; Vial 4: 10 mL sterile water; Vial 5: 1 mL sterile EtOH (99%); Vial 6: 10 mL sterile isotonic saline (NaCl 0.9% solution in water); RBF: 70 mL sterile water, HPLC column: Phenomonex Luna C18, 250×10 mm, mobile phase: 45% sterile EtOH (99%) in 55% NaH_2PO_4 (70 mM).

Synthesis on TRACERlab FX C Pro

The [^{11}C]CO_2 is delivered from the cyclotron by nitrogen pressure *via* a Tefzel delivery-line and trapped at room temperature in a column packed with molecular sieves (4 Å) and a nickel catalyst (Nickel on Shimalite, Shimadzu Corporation), *c.f.*, Figure S1. The [^{11}C]CO_2 is reduced to [^{11}C]CH_4 by heating the column (375 °C, 30 s) and is passed through a water trap (Ascarite II 20-30 mesh and P_2O_5 with Sicapent moisture indicator) by a 120 mL/min stream of He gas (Air Liquide Denmark A/S). The [^{11}C]CH_4 is retained on a Carbosphere trap cooled to -130 °C with liquid N_2. The Carbosphere column is heated slowly to 80 °C and gaseous [^{11}C]CH_4 is released into a circulation loop, which includes a gas pump, an I_2 column (100 °C), an I_2 reactor tube (740 °C), two Ascarite II (20-30 mesh) columns (absorbs hydrogeniodide, HI), and a Porapak Q column at room temperature. The porapak Q traps the produced [^{11}C]CH_3I while unconverted [^{11}C]CH_4 is recycled back for reaction with I_2. At end of recirculation, the [^{11}C]CH_3I is released by heating the Porapak Q (190 °C)

and passed through a pre-heated AgOTf column (200 °C) to produce [^{11}C]CH_3OTf and trapped in the precursor solution (-20 °C).

An external reaction vessel is used for the production of [^{11}C]PiB, which consists of a glass vial and a 11 mm white silicone/red Polytetrafluoroethylene (PTFE) cap, *c.f.*, Figure S1 and S3. The external reaction vessel was introduced due to repeatedly low yields of [^{11}C]PiB and lack of robustness of the synthesis. A long green needle is attached to the outlet of V8 and placed in the trapping solution in the reaction vial, and a small orange needle is used as a vent (Figure S1). Upon maximal trapping of radioactivity, the needles are removed, and the reaction vessel is heated to 50 °C for 2.5 min. At end of reaction, the reaction vessel is cooled to 40 °C. Subsequently, a long needle is attached to the outlet of V3 and placed in the reaction vessel along with a small venting needle. The reaction mixture is diluted with sterile water (1 mL, Vial 3). The vent needle is then removed and the V8 needle is again placed into the reaction vessel.

Then a helium flow is used to lead the crude product onto the HPLC loop, *c.f.*, Figure S1 and S3. The crude is then injected and purified using a semipreparative reversed phase HPLC (flow rate: ~6 mL/min) and the product (retention time: ~7-8 min) is collected in a round bottomed flask containing sterile water (70 mL). The diluted [^{11}C]PiB is then passed through a preconditioned (10 mL EtOH followed by 10 mL sterile H_2O) Sep-Pak C18 Plus Short to remove excess EtOH from the final product. The Sep-Pak is washed with sterile water (10 mL, Vial 4) and eluted to a product flask with sterile EtOH (1 mL, Vial 5) followed by sterile isotonic saline (10 mL, Vial 6). The 10-11 mL final formulation is then passed through a sterile (Millex-GV 0.22 µm, SLGV033RS) filter into a sterile dose vial to afford 1.98±1.00 GBq of [^{11}C]PiB. A sample (0.3 mL) is taken for QC analysis prior to product release.

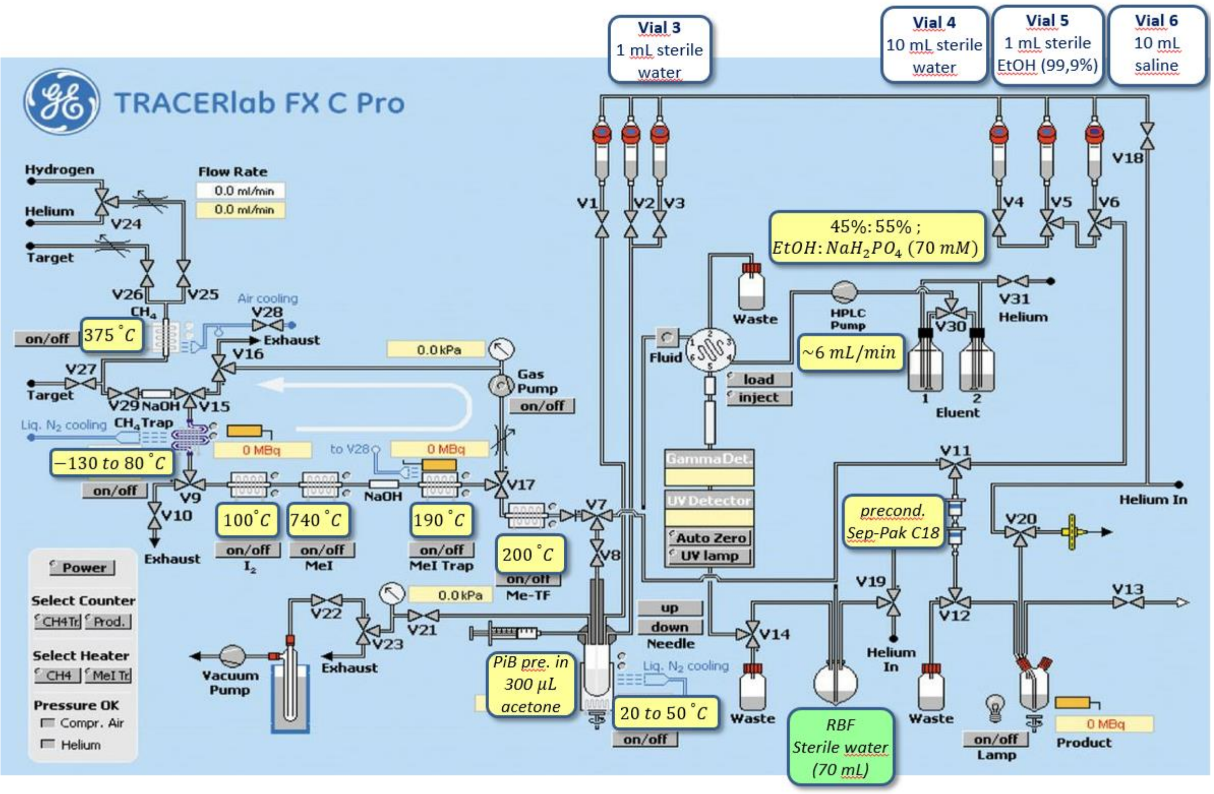

Figure S1: Scheme of the TRACERlab FX C Pro synthesis module software and conditions for [^{11}C]PiB synthesis at AUH.

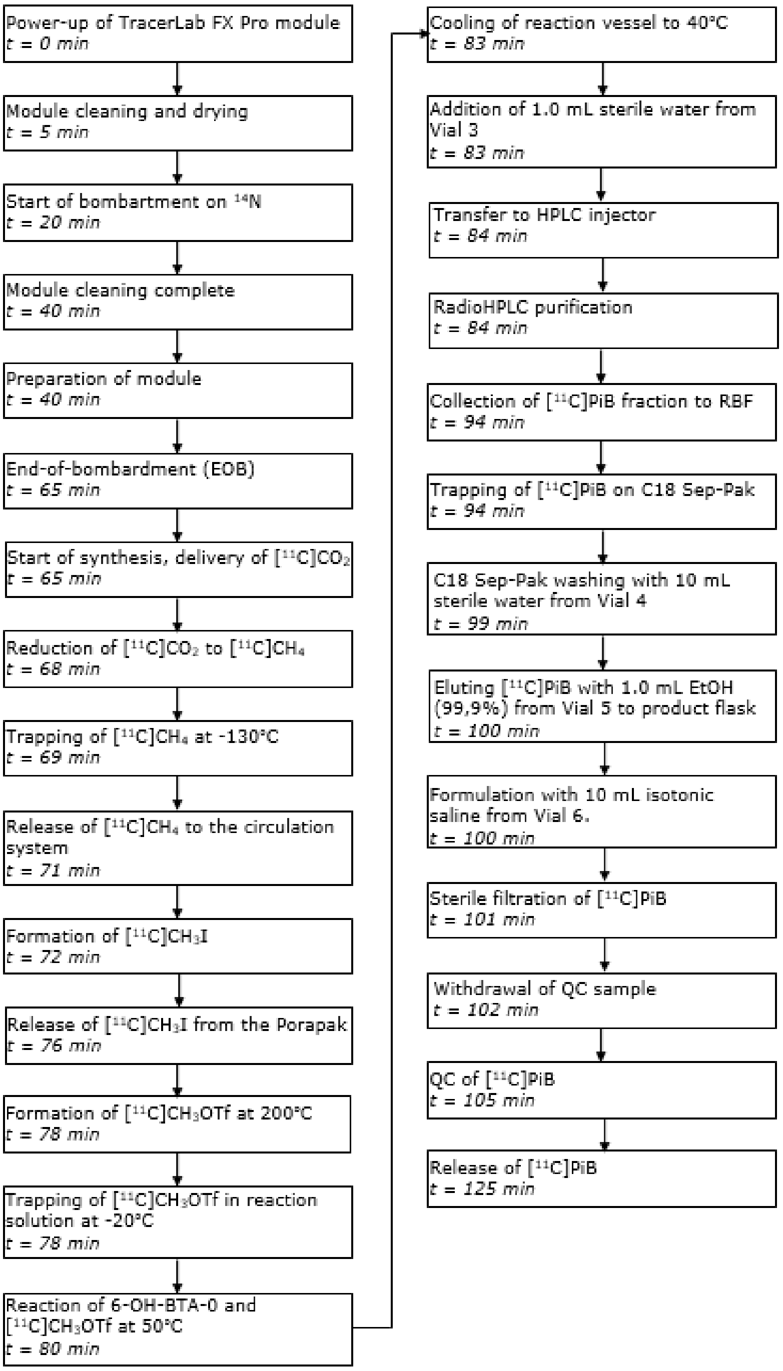

Figure S2: Overall [^{11}C]PiB synthesis flowchart at AUH.

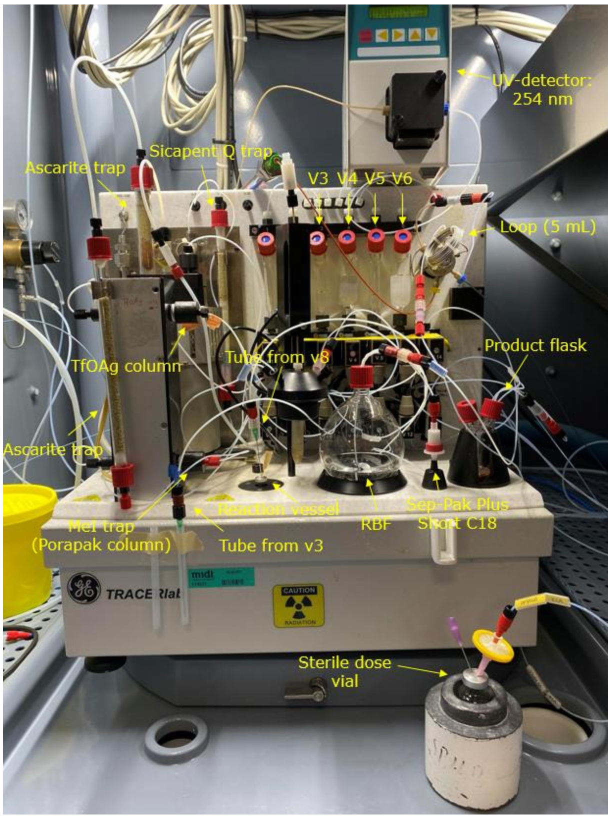

Figure S3: TRACERlab FX C Pro setup for [^{11}C]PiB synthesis at AUH.

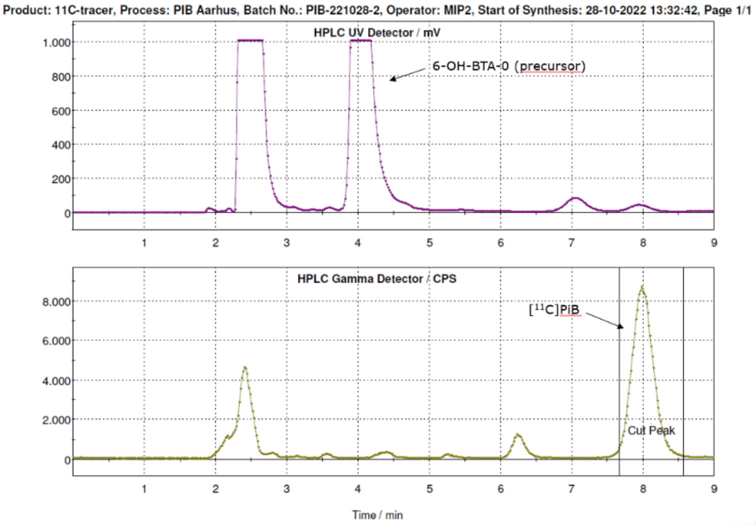

Figure S4: Semi-preparative HPLC traces for [^{11}C]PiB production at AUH.

S2: Radiosynthesis of [^{11}C]PiB at HUH

The [^{11}C]PiB radiosynthesis at HUH is carried out on a TracerMaker module (see Fig. S5) by use of [^{11}C]CH_4 produced on an IBA Cyclone 18/18 cyclotron via. $^{14}N(p,\alpha)^{11}C$ nuclear reaction of 18 MeV protons on 95% N_2 + 5% H_2 target gas (Air Products). Bombardment of the target gas is performed with an irradiation time of 25-30 min and beam current of 27 μA (Table 3, main text). The overall production is shown as a flowchart in Fig. SX.

Preparation and Synthesis on TracerMaker

Prior and after each production, the TracerMaker module and the HPLC loop is cleaned and dried with EtOH and helium, respectively. This procedure is performed by an automated sequence on the module. Preparation of the [^{11}C]PiB production are as follows (see Fig. S6 for schematic overview and S7 for production flowchart). The synthesis program is initiated, single-use consumables from previous productions are removed, and waste-container and EtOH-bottle (99.8%, >200 mL, V21) levels are checked. At this point, the system automatically sets the temperatures of gas-traps and ovens: I_2 oven (60 °C), quartz oven (720 °C), HayeSep (-125 °C), and AgOTf oven (200 °C). Sterile water bottle is replaced (100 mL, V14), liquid N_2 is loaded into the build-in Dewar-flask (for cooling gas-traps), and an empty 3.5 mL reactor vial (3.5-HRSV, Thermo Scientific) is placed into reactor 2 (part of cleaning process, discarded before synthesis), *c.f.*, Figure S6. While the system initializes and cleans, the HPLC eluent A for product separation is prepared. This is done by addition of 2.2 g L-ascorbic acid and 0.5 mL H_3PO_4 () to 500 mL sterile water bag (AE0303, Baxter). Eluent A is then mounted to the HPLC, eluent B level is checked (99.8% EtOH, >300 mL) and the HPLC systems pressure is checked while under operation. The precursor (6-OH-BTA-0, 1.0 mg) is dissolved in dry 2-butanone (150 μL) and transferred to an empty reactor vial and mounted in reactor

2 prior to synthesis. Note: the empty reactor vial is “dried” by washing with dry 2-butanone (approx. 0.5 mL) before the precursor solution is added.

The [^{11}C]CH_4 is delivered from the cyclotron by nitrogen pressure (300 mL/min) and trapped on a HayeSep column (-125 °C), and briefly flushed with helium (500 mL/min, 20 sec) to remove residual gas. The [^{11}C]CH_4 is then released (HayeSep heated to -10 °C) and converted to [^{11}C]CH_3I by cycling through a loop for 5 min with a rate of 900 mL/min, which consists of a gas-pump, I_2 column, and quartz oven fitted with a trap (Ascarite and P_2O_5) for capturing HI, *c.f.*, Fig. S6. The [^{11}C]CH_3I is trapped on the HayeSep column during cycling and excess [^{11}C]CH_4 is removed by flushing the loop with helium (500 mL/min, 40 sec; followed by 100 mL/min, 10 sec).

The [^{11}C]CH_3I is then released by heating the HayeSep column (200 °C) and converted to [^{11}C]CH_3OTf by passed through an AgOTf column (200 °C) by helium flow (100 mL/min, 2 min). Note: Heating of the AgOTf column is automatically initialized at start of the synthesis software and runs for approx. 25-35 min prior to [^{11}C]CH_3I conversion. The [^{11}C]CH_3OTf/Helium flow is bubbled directly into the reactor vial (reactor 2, -10 °C). The mixture in reactor 2 is then heated to 85 °C and allowed to react for 2.5 min, before quenched with eluent A (2.5 mL) and transferred to the HPLC injection loop. The reactor vial is then washed with additional eluent A (0.5 mL) and the wash is transferred to the HPLC injection loop.

HPLC separation and formulation

Separation of [^{11}C]PiB from impurities are automatically performed on a HPLC system (Fig. S5 and S6) consisting of two Knauer Smartline 100 pumps and a Kinetex@ 2.6 µm C18 100 Å column (50×4.6 mm, Phenomenex), with a flowrate 8 mL/min and eluent ratio of A:B (0.1% H_3PO_4(aq):EtOH) = 70:30. Eluent A additionally contains 25 mM *L*-ascorbic acid (*vide supra*) to prevent radiolysis of the product. In the beginning of chromatogram (Fig. S8), an impurity is

detected by the HPLC radiodetector (GM7, Fig. S6) and is eluted directly to the waste container. After a 180 s timer, the system is programmed to automatically collect the [^{11}C]PiB fraction when the input from the HPLC radiodetector reaches 700 counts/s. The [^{11}C]PiB fraction is collected for 20 sec (2.67 mL) into a 30 mL empty sterile vial. This is further transferred through a sterile filter (Millex-GS 0.22 µm, SLGSV255F) into the product vial containing 9 mL PBS to afford 0.83±0.29 GBq of [^{11}C]PiB. For the QC procedure, a 0.25 mL sample is collected for analysis prior to release.

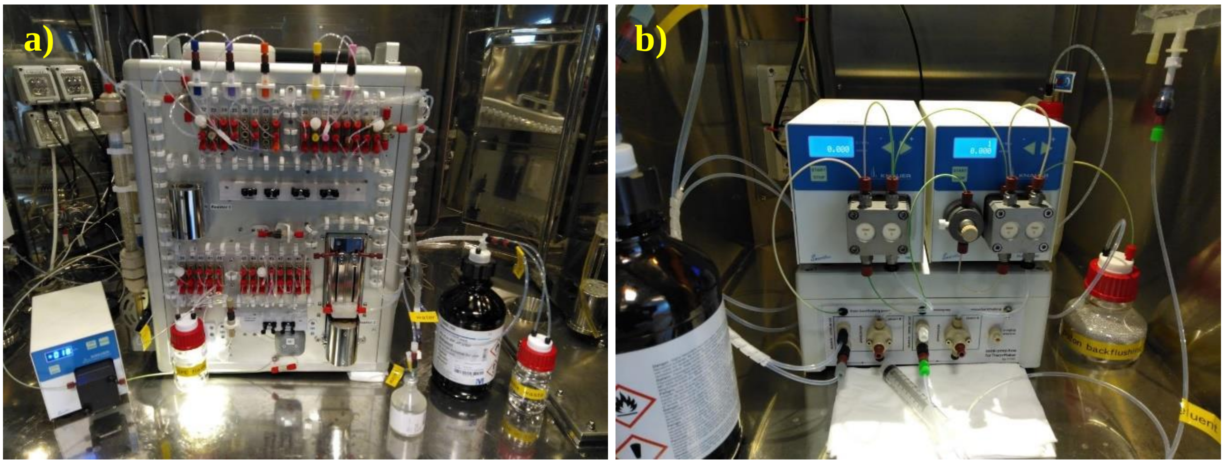

Figure S5: a) TracerMaker setup for [^{11}C]PiB synthesis at HUH. **b)** HPLC pumps beneath hot-cell for separation and formulation of [^{11}C]PiB.

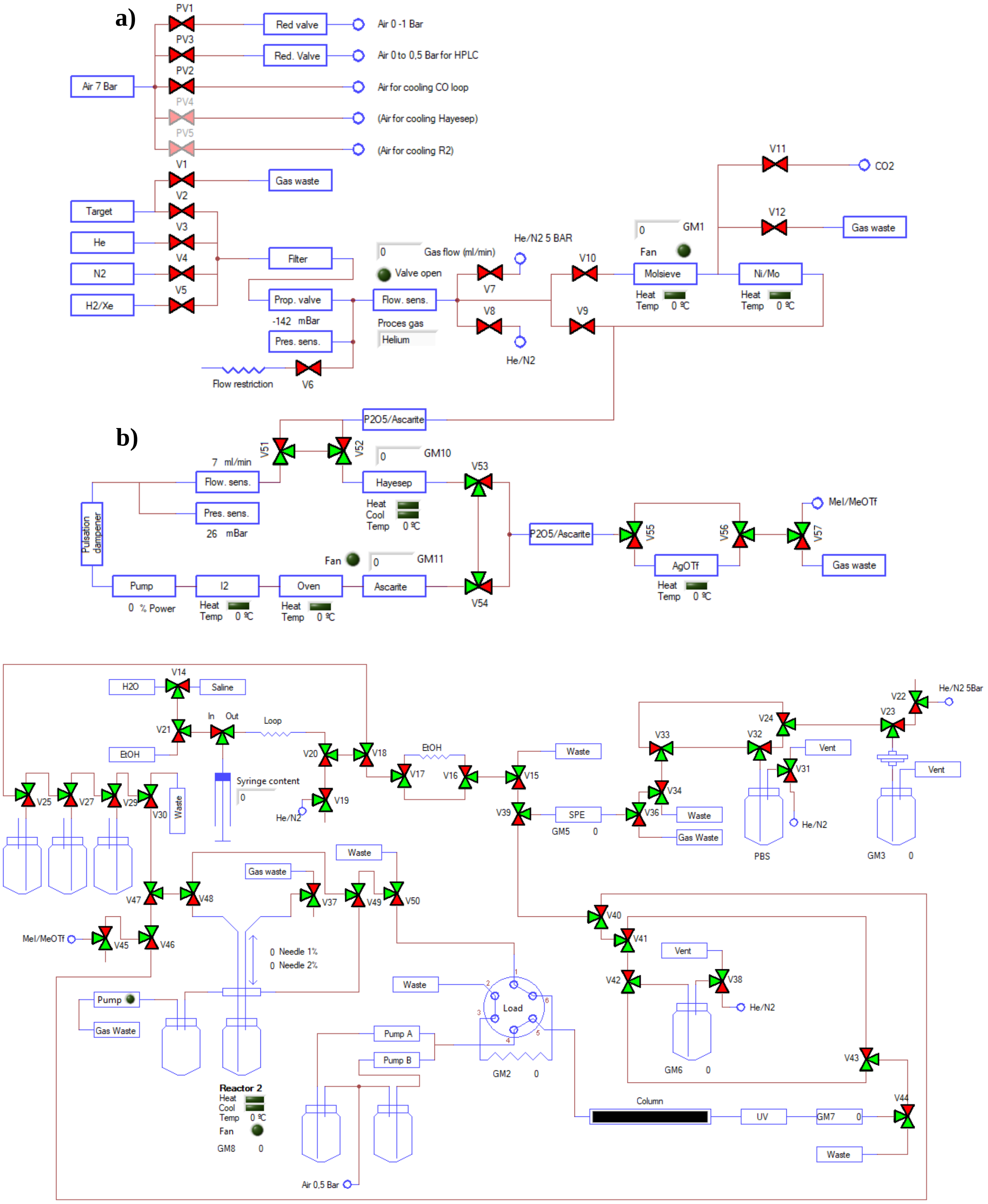

Figure S6: Schematic overview of the TracerMaker setup at HUH, with **a)** dry and **b)** wet parts of the system.

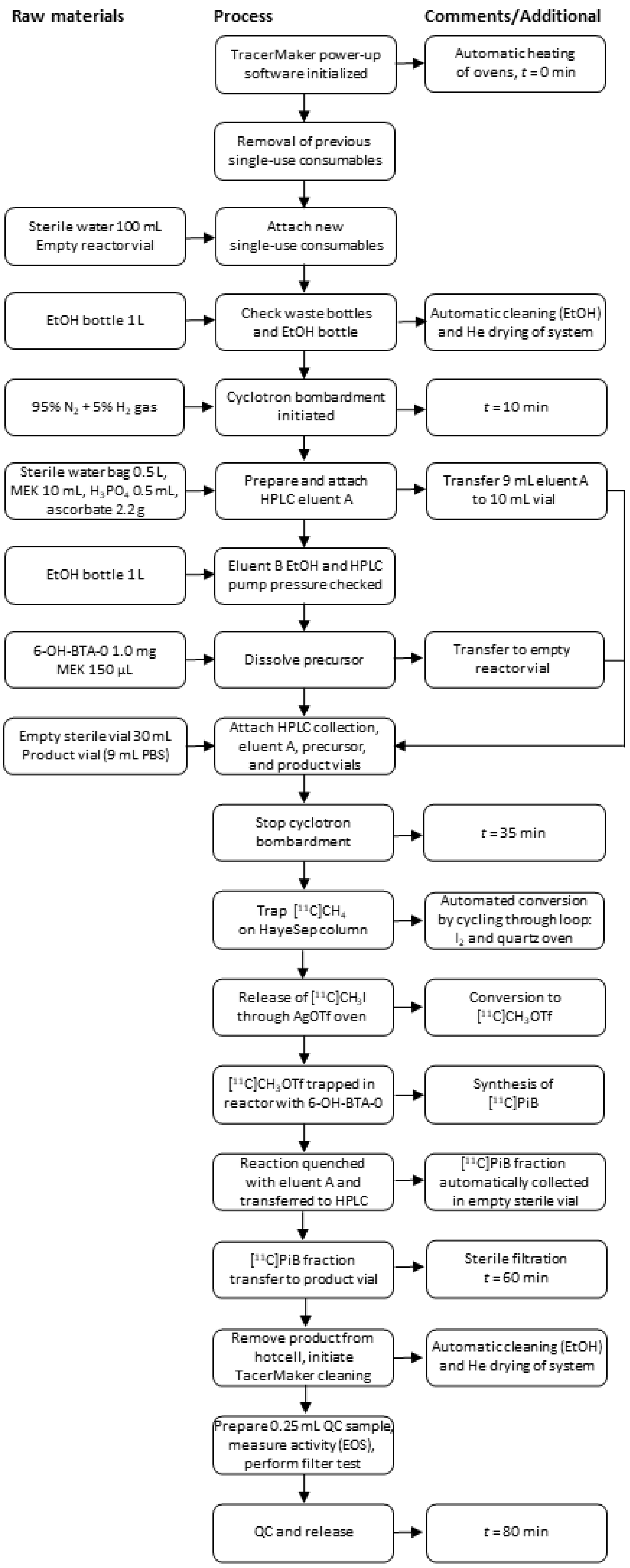

Figure S7: Overall [^{11}C]PiB synthesis flowchart at HUH. Note: MEK (methyl ethyl ketone) is 2-butanone and ascorbate is *L*-ascorbic acid.

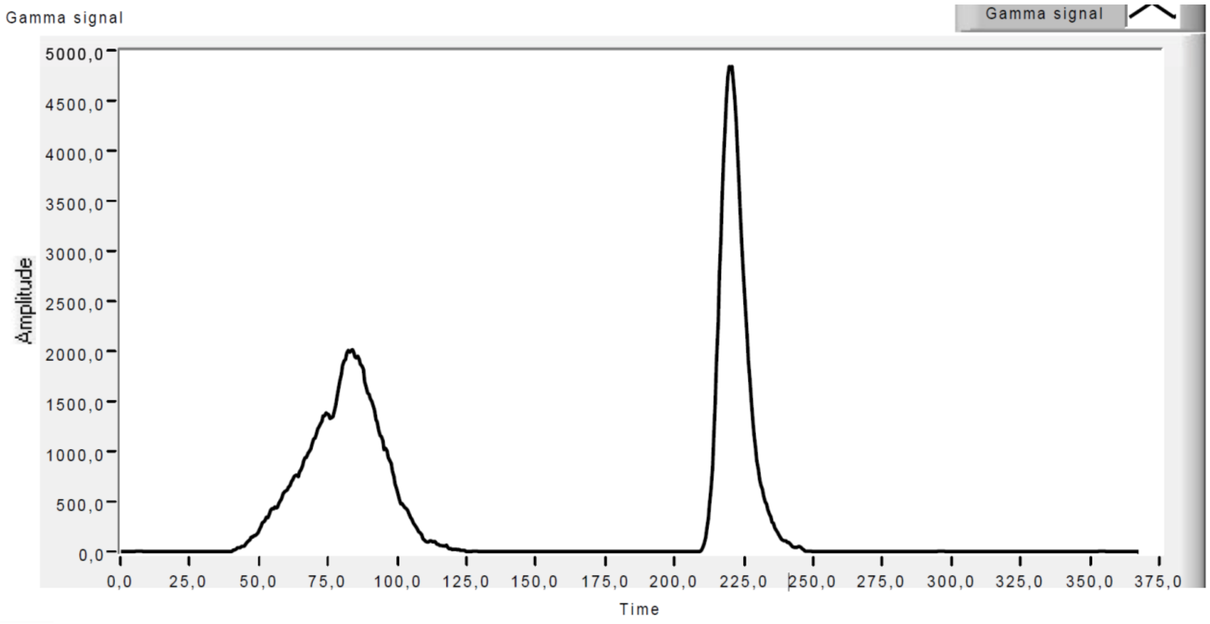

Figure S8: Chromatogram (gamma radiodetector, GM7) of [^{11}C]PiB product isolation by semi-preparative HPLC at HUH, where the first signal is an radioactive impurity assigned to [^{11}C]CH_3OH and the second signal is [^{11}C]PiB.

S3: Radiosynthesis of [^{11}C]PiB at OUH

At OUH, [^{11}C]PiB is produced on a TRACERlab FX C Pro automated synthesis module by the use of [^{11}C]CO_2. See Fig. S9 for a schematic overview in the TRACERlab software and Fig. S10 for a flowchart of the overall synthesis. [^{11}C]CO_2 is produced using a GE PETtrace 800 employing a target gas mixture comprised of 99.5% N_2 and 0.5% O_2 (Strandmøllen A/S, Klampenborg). Duration of the irradiation times is ranging from 30 to 60 minutes with beam currents varying between 50 and 60 μA (Table 3, main text).

Preparation and production on TRACERlab FX C Pro (GE HealthCare)

The TRACERlab FX C Pro synthesis module and the HPLC loop are cleaned by two iterations of the following procedure. Loading of acetone (3 mL) into vial 3, which is then used to wash the fluid path though the reactor and injector. The fluid path is subsequently dried using a helium flow. The nickel catalyst within the TRACERlab FX C Pro synthesis module is conditioned using a continuous flow (20 mL/min) of hydrogen (obtained from Strandmøllen A/S, Klampenborg) at a temperature of 350 °C for a minimum of 10 minutes. The module is allowed to cool to ambient temperature before use.

Simultaneously, the HPLC column (specifically, the Merck Chromolith Performance RP-18e, sized at 100×10 mm) is conditioned using the mobile phase, consisting of 25% EtOH in 15 mM phosphoric acid in WFI, with a flow rate of 5 mL/min for a minimum duration of 10 minutes. Finally, the PIB synthesis is readied by loading of 6-OH-BTA-0 (1.0 ± 0.1 mg) dissolved in acetone (300 μL) into the reaction vial and filling vial 3 with 0.5 mL of a 15 mM phosphoric acid solution. Prior to synthesis start liquid nitrogen is loaded into the Dewar.

The synthesis of [^{11}C]PiB starts with converting [^{11}C]CO_2 into [^{11}C]CH_3OTf by using chemicals specified by the manufacturer (GE HealthCare) of the TRACERlab FX C system. The [^{11}C]CO_2 delivered from the target is trapped in the methane oven and reduced to [^{11}C]CH_4 in the

presence of H_2 gas (Strandmøllen A/S, Klampenborg). The resulting $[^{11}C]CH_4$ is then transferred to a liquid N_2 cold-trap (-100 °C) by a stream of helium. The cold trap consists of Carbosphere. The trapped $[^{11}C]CH_4$ is released by heating the cold-trap to 80 °C and the $[^{11}C]CH_4$ is then circulated in a gas conversion loop passing over iodine at 700 °C, reacting to form $[^{11}C]CH_3I$, which is collected on a Porapak cartridge. To convert $[^{11}C]CH_3I$ to $[^{11}C]CH_3OTf$, the AgOTf is heated on Carbopac to 200 °C and the newly formed $[^{11}C]CH_3I$ is passed through the material directly into the labeling solution. The $[^{11}C]CH_3OTf$ is captured in a labeling solution composed of 6-OH-BTA-0 (1.0 mg) and acetone (300 µL) at 5 °C. Once all the activity is transferred to the reactor, the temperature is increased to 60 °C for 120 sec.

HPLC separation and formulation

The reaction mixture is diluted using a 15 mM H_3PO_4 solution and injected onto the integrated preparative HPLC system of the TRACERlab FX C Pro (Pump: Sykam S1021, UV detector: Knaur K-2001) fitted with a Merck Chromolith Performance RP-18e (100×10 mm) column, running isocratically with an eluent composed of 25% EtOH in 15 mM phosphoric acid in WFI at a flow of 6.5 mL/min. The cut peak is collected after approximately 8 minutes into a PBS buffer vial. The final product is sterilized by cannulation of the solution into a sterile vial through a Millex-GV filter (0.22 µM PVDF, SLGV0250S) to afford 3.17 ± 1.2 GBq of $[^{11}C]$PiB. A sample (0.5 mL) is taken for QC analysis prior to product release.

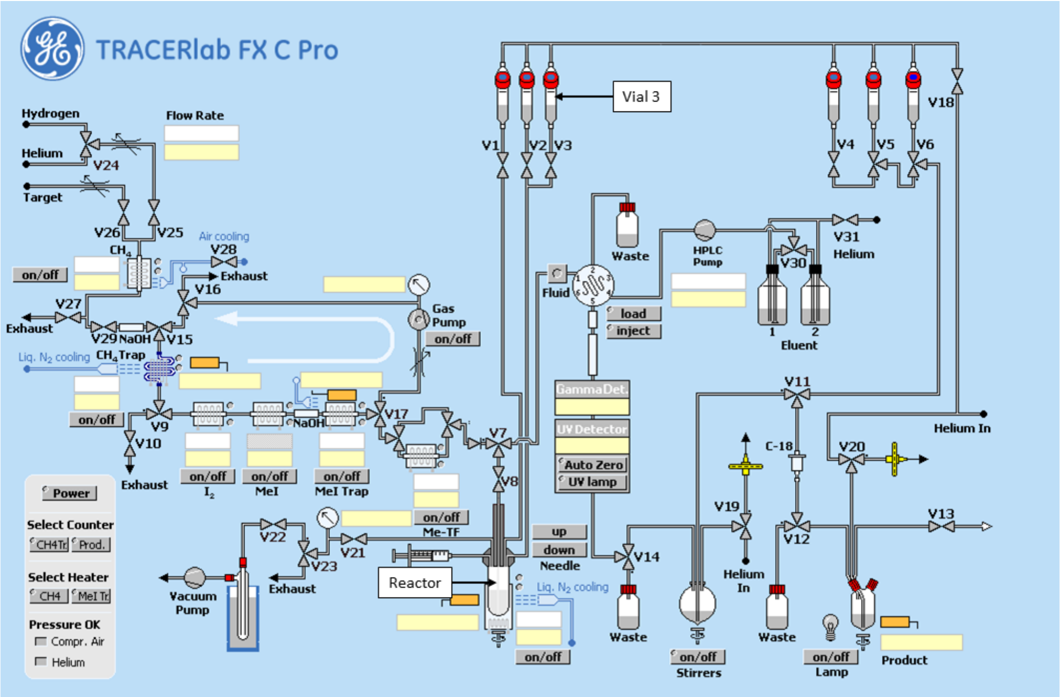

Figure S9: Schematic overview of TRACERlab FX C Pro setup at OUH.

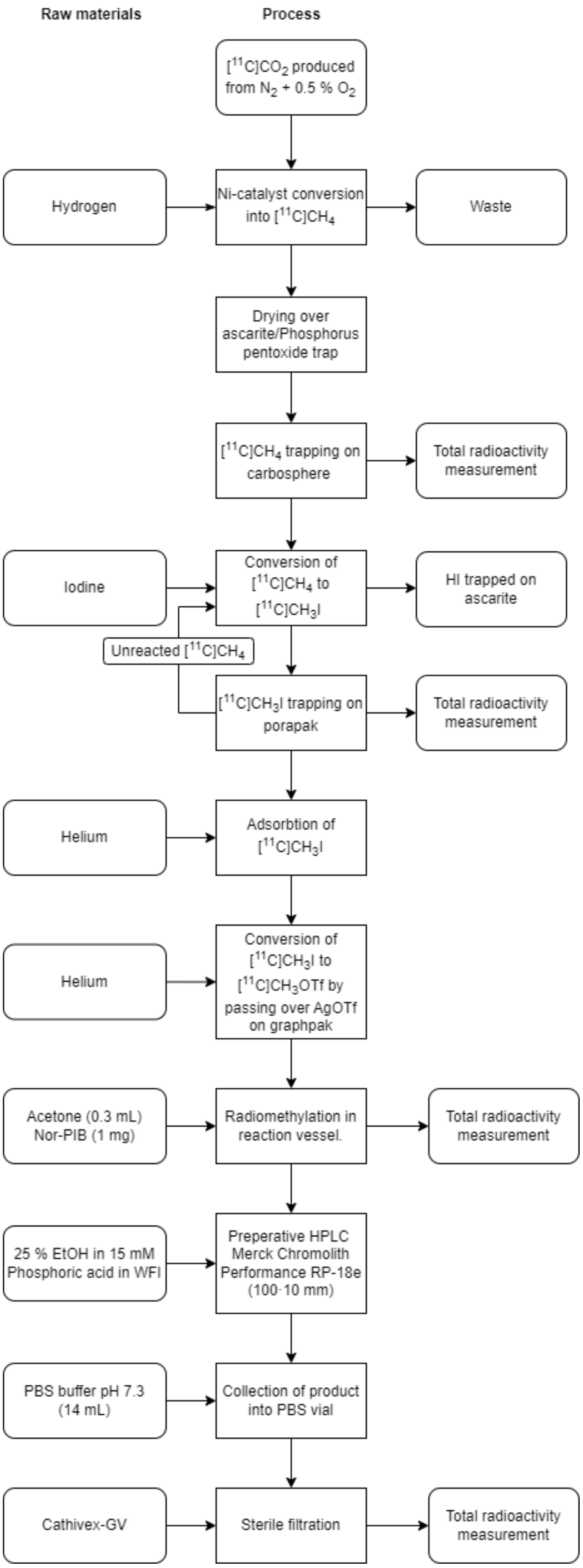

Figure S10: Overall [^{11}C]PiB synthesis flowchart at OUH.

S4: Radiosynthesis of [^{11}C]PiB at RH

At RH, the [^{11}C]PiB radiosynthesis is performed on either a ScanSys or a TracerMaker module (see Figs. S11 and S12). The [^{11}C]CO_2 and [^{11}C]CH_4 for synthesis are produced in the target by $^{14}N(p,\alpha)^{11}C$ of nuclear reaction of 16 MeV protons on 99.5% N_2 + 0.5% O_2 (CTI XP cyclotron) or 95% N_2 + 5% H_2 (Scanditronix cyclotron) target materials, respectively. Bombardment of the target gas is performed with an average irradiation time and beam current of 44.5 min and 52.7 μA, respectively (Table 3, main text). However, [^{11}C]CO_2 is preferred for the production, as [^{11}C]CH_4 is observed to give drastically lower [^{11}C]PiB yields together with a large byproduct (product:byproduct ratio ≈ 1:8) in the beginning (1.0-1.5 min) of the preparative HPLC.

Preparation and Synthesis on ScanSys

The ScanSys system is cleaned after each synthesis by an automatic sequence by flushing the gas part (Schematic overview in Fig. S13) with He, and rinse of the wet part (Fig. S13b) with 70% EtOH. Prior to production, the synthesis module and cyclotron target are flushed (15 min) with He and target gas, respectively. The synthesis program is initiated, Dewar-flask is filled with liquid N_2, and 96% EtOH and water bottle levels are checked (Figs. S11 and S13). At this point, the system automatically sets the temperatures of gas-traps and ovens: I_2 oven (RT → 60 °C °C), quartz oven (760 °C), AgOTf oven (200 °C), HayeSep (-180 RT → 10 °C), Ni oven (350 °C), and molecule sieve trap (<30 °C). The HPLC eluent (500 mL 30:70 EtOH:0.1% H_3PO_4, 25 mM ascorbic acid) flask is connected, and waste-bag emptied and reconnected. Then 4.0 mL aqueous 0.1% H_3PO_4, 25 mM ascorbic acid is transferred to a 5 mL V-shaped vial and placed in rack position 7 (Fig. S13), and 5.0 mL acetone is added to a 7 mL vial and placed in rack position 5 (Fig. S13). Then, the HPLC pumps are primed, the pressure is checked (approx. 50-60 bar), and the product formulation 30 mL vial with 9 mL PBS buffer (2% phosphate buffer in saline, pH = 7) is connected to outlet 3 via. a

sterile filter (Millex-GV 0.22 μm, SLGV033RS). The reaction vial, which contains precursor (1 mg) is dissolved in 2-butanone (300 μL), is placed in reactor 2 (position 13, Fig S13).

The $[^{11}C]CO_2$ is delivered from the cyclotron via. He through a 100 m long, 1/16-inch OD stainless steel tube and is converted to $[^{11}C]CH_4$ by catalytic reduction via Ni-catalyst by H_2 gas (6.0, Strandmøllen, Denmark) at 350 °C. The HayeSeptrap is then flushed with He and $[^{11}C]CH_3I$ is produced by gas phase iodination of $[^{11}C]CH_4$ by circulation (flow rate: 800-1000 mL/min) through the HayeSep column 10 °C) and I_2 oven loop. The system is flushed with He to remove unreacted $[^{11}C]CH_4$. The $[^{11}C]CH_3I$ is then released by heating the HayeSep (200 °C) and converted to $[^{11}C]CH_3OTf$ via. the AgOTf oven. The produced $[^{11}C]CH_3OTf$ is collected in the reaction vial (reactor 2). The mixture is heated to 80 °C for 3 min before diluted and transferred to the semi-preparative HPLC system for purification and formulation.

HPLC separation and formulation on ScanSys

The preparative HPLC system consists of a Knauer 100 pump, Knauer UV 120 detector coupled in series with a radioactivity detector, Gilson 401 dilutor, Cavarno XE 1000 syringe pump and Rheodyne 7750E06 injection valve with 5 mL loop. The reaction mixture (300 μL) is diluted with 4.5 mL HPLC eluent buffer (0.1% H_3PO_4, 25 mM *L*-ascorbic acid) and subsequently injected onto an Onyx Monolithic semi-PREP C18 HPLC column (10×100 mm, Phenomenex). Eluent: 96% EtOH:0.1% H_3PO_4, 25 mM ascorbic acid (30:70); flow rate 6.0 mL/min; wavelength λ = 254 nm. The product fraction is collected within 1 min (6.0 mL) and passed through the sterile Millex-GV filter into the 30 mL sterile vial containing 9.0 mL PBS buffer to afford 1.46±0.64 GBq of $[^{11}C]$PiB in approx. 11% (EOB) yield. The final solution for injection contains 10 m/m% EtOH. A sample (0.5 mL) is submitted to QC analysis prior to product release.

Preparation and Synthesis on TracerMaker

The TracerMaker system is cleaned prior to and after each synthesis by an automatic sequence by flushing the gas part (See Fig. S14 for schematic overview) with He, and rinse of the wet part (Fig. S14b) with 70% EtOH. The HPLC eluent (500 mL 30:70 EtOH:0.1% H_3PO_4, 25 mM ascorbic acid) is connected to HPLC pump 1 and the built-in Dewar-flask is filled with liquid N_2. The synthesis program is initiated and single-use consumables from previous productions are removed. At this point, the system automatically sets the temperatures of gas-traps and ovens: I_2 oven (RT → 60°C), quartz oven (760 °C), AgOTf oven (200 °C), HayeSep (-160 °C), Ni oven (350 °C), and molecule sieve trap (RT → 300 °C → 30 °C). Then, a 4 mL reactor vial is filled with 96% EtOH and placed in reactor 2 (Fig. S14). The acetone- and EtOH-flask levels are checked, and the HPLC pressure is checked. The product line is rinsed with 70% EtOH by connecting it to an empty waste vial while the flow is observed. The product line is then connected to a product vial containing 9 mL PBS buffer via. a sterile filter (Millex-GV 0.22 µm, SLGV033RS). The precursor (1 mg, ABX, 5101.0001) is dissolved in 2-butanone (300 µL) and transferred to a 4 mL vial, which is placed in reactor 2 (Fig S14). Then a 4 mL vial is prepared with 0.2 mL EtOH and 2.3 mL 0.1% H_3PO_4, 25 mM ascorbic acid, and placed at position D (Fig. S14).

The $[^{11}C]CO_2$ is delivered from the cyclotron via. He (300 mL/min) and trapped on the molsieves. The $[^{11}C]CO_2$ is reduced to $[^{11}C]CH_4$ on the Ni-catalyst by H_2 gas (6.0, Strandmøllen, Denmark) at 350°C and the system is subsequently flushed with He. The gas phase iodination of $[^{11}C]CH_4$ is performed by circulation (flow rate: 900 mL/min) through the HayeSep column (-10 °C) and I_2 oven loop. The system is flushed with He to remove unreacted $[^{11}C]CH_4$. The $[^{11}C]CH_3I$ is released from the HayeSep (200 °C) and converted to $[^{11}C]CH_3OTf$ via. the AgOTf oven. The $[^{11}C]CH_3OTf$ is then collected in the reaction vial at-10 °C (reactor 2). The mixture is heated to 80 °C

for 3 min before diluted (solution at position D) and transferred to the semi-preparative HPLC system for purification.

HPLC separation and formulation on TracerMaker

The preparative HPLC system consists of a Knauer P.4.1S pump, Knauer Azura UVD 215 detector coupled in series with a radioactivity detector and Rheodyne 7750E06 injection valve with 5 mL loop. The reaction mixture (300 µL) is diluted with 2.3 mL HPLC eluent + 0.2 mL 96% EtOH and subsequently injected onto an Onyx Monolithic semi-PREP C18 HPLC column (10×100 mm, Phenomenex). Eluent: 96% EtOH:0.1% phosphoric acid, 25 mM ascorbic acid (30:70); flow rate 6.0 mL/min; wavelength λ = 370 nm. The product fraction is collected within 1 min (6.0 mL) and passed through the sterile Millex-GV filter into the 30 mL sterile vial containing 9.0 mL PBS buffer (final solution for injection contains 10% EtOH) to afford 1.46±0.64 GBq of [^{11}C]PiB in approx. 11% (EOB) yield. A sample (0.5 mL) is submitted to QC analysis prior to product release.

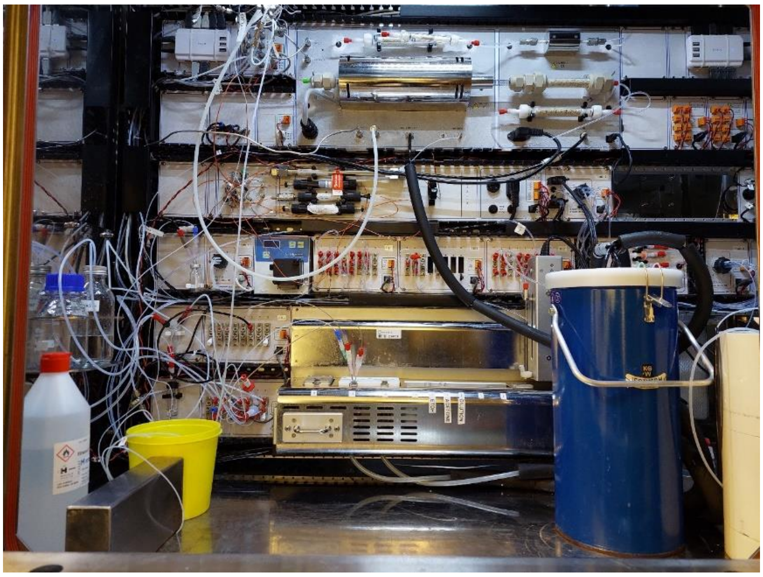

Figure S11: ScanSys setup for [^{11}C]PiB synthesis at RH.

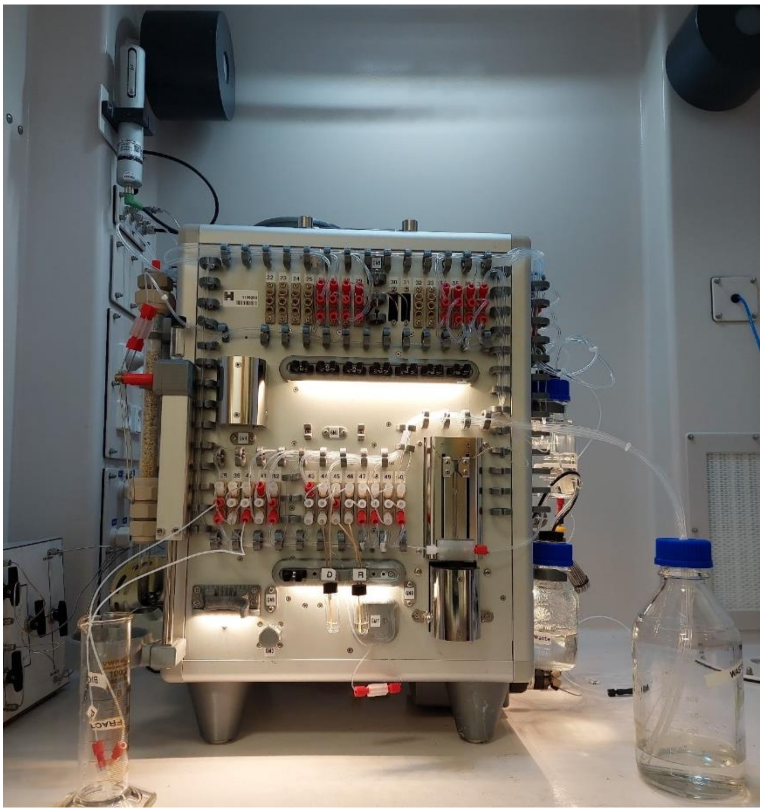

Figure S12: TracerMaker setup for [^{11}C]PiB synthesis at RH.

a)

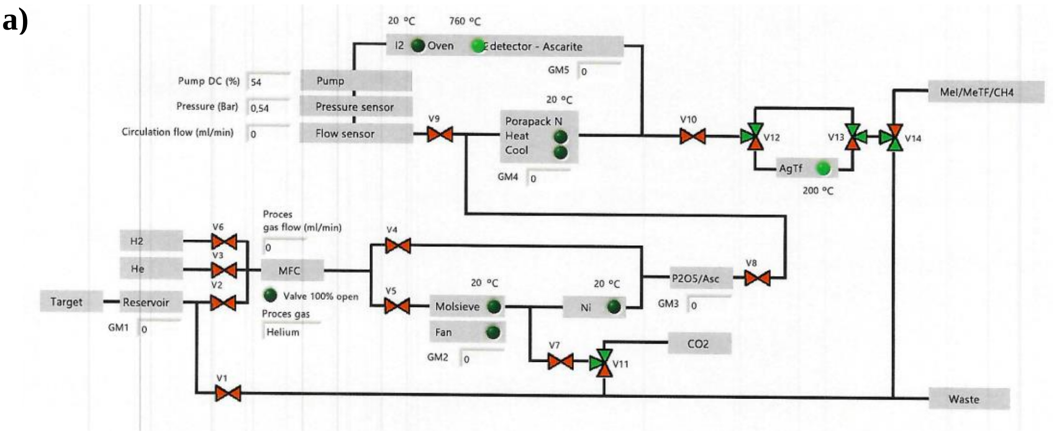

b)

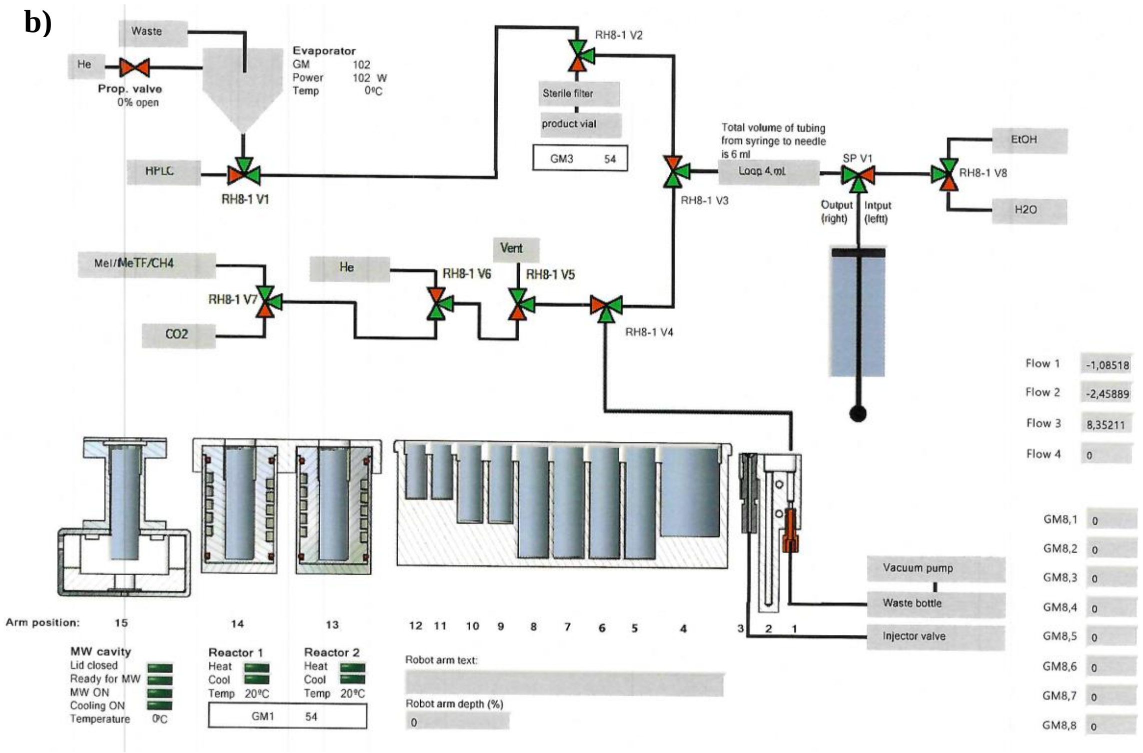

Figure S13: Schematic overview of the ScanSys setup at RH, with **a)** dry and **b)** wet parts of the system.

a)

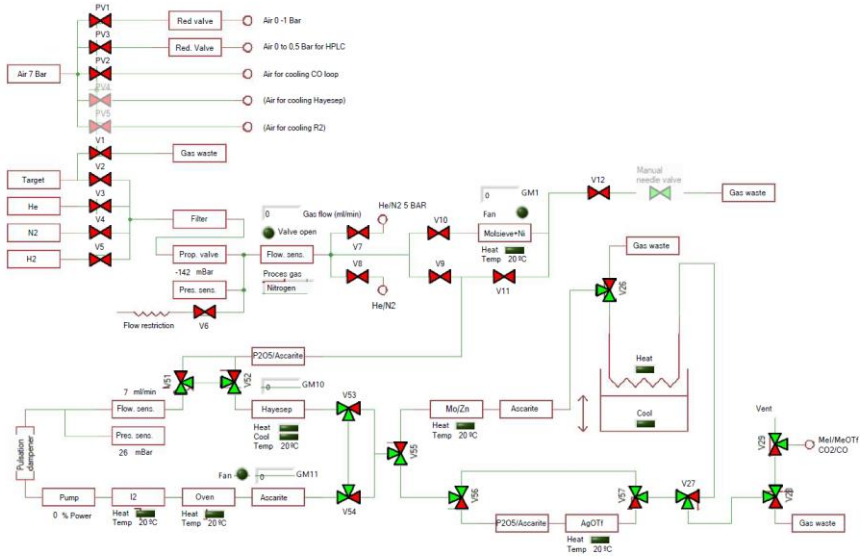

b)

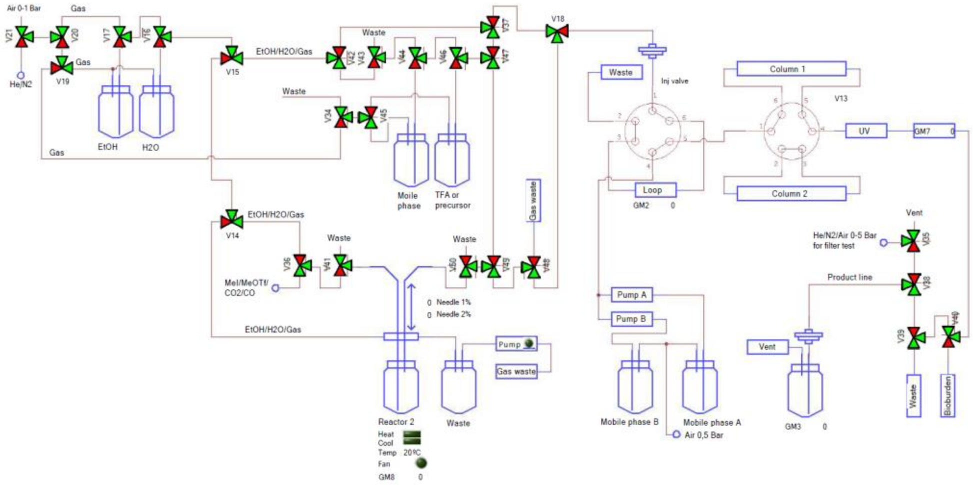

Figure S14: Schematic overview of the TracerMaker setup at RH, with **a)** dry and **b)** wet parts of the system.

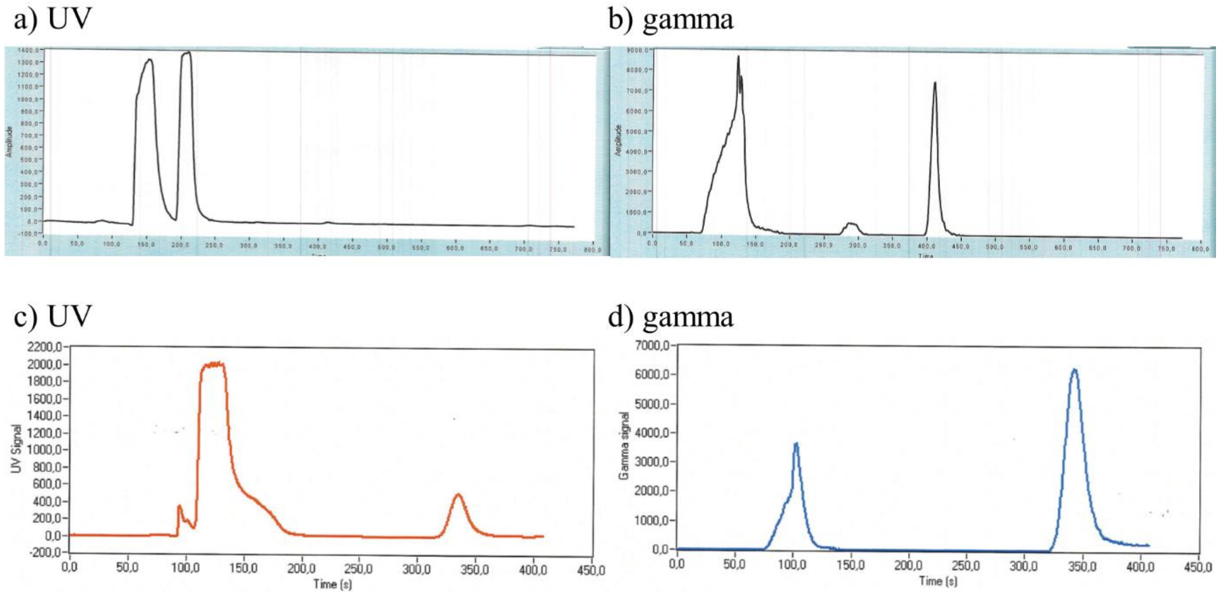

Figure S15: Representative semi-preparative HPLC traces for [^{11}C]PiB productions at RH; where **a)** is UV and **b)** radioactive traces for production with [^{11}C]CH_4, and **c)** UV and **d)** radioactive traces for production with [^{11}C]CO_2.

S5: Quality Control and Specifications for [^{11}C]PiB

The specifications for the final [^{11}C]PiB product are summarized in Table S5 and the quality control equipment and parameters are summarized in Table S6. For the QC procedure, HPLC is used to test for precursor (6-OH-BTA-0) and [^{11}C,^{12}C]PiB concentrations as wells as radio chemical purity (RCP), *i.e.*, the radioactivity originates from [^{11}C]PiB and no other radioactive compounds. The GC is used to test for residual solvent (2-butanone or acetone) as well as excipient EtOH concentrations. The total radioactivity (yield) is measured by dose calibrators. The radioactive yield is then used to calculate A_s and/or A_m in regard to the specifications (Table S5). The pH is tested on a calibrated pH-meter and appearance is performed by visual inspection. Endotoxin levels are in all cases performed after release on the respective equipment from Charles River. Unknown impurities and the highest individual unknown impurity are determined by HPLC (together with that of free precursor and [^{11}C/^{12}C]PIB concentrations.

Table S1: Final [^{11}C]PiB product specifications at the four production sites, AUH, HUH, OUH, and RH. Where *V* is the total volume of the final product, A_s is the specific activity, A_m is the molar activity, $C_{6\text{-OH-BTA-0}}$ is concentration of the precursor, C_{PiB} is the total concentration of both [^{11}C]PiB and cold [^{12}C]PiB, RCP is the radiochemical purity, and γ-spec. is gamma-ray spectroscopy.

	**AUH**	**HUH**	**OUH**	**RH**
V [mL]	10±1	10.75±1.25	20±1	15±1
Yield [MBq]	200-3000	95-1440	50-5720	≤ 3500
A_m [GBq/μmol]	--	> 11	> 10	--
pH	4.0-7.0	6.0-7.5	4.5-8.5	4.5 < pH < 8.5
$C_{6\text{-OH-BTA-0}}$ [μg/mL]	< 5	≤ 0.2	< 100/*V*	< 1.0
C_{PiB} [μg/mL]	< 15	≤ 1.0	< 100/*V*	< 1.0
RCP [%]	> 95	> 95	≥ 95	≥ 95
2-butanone [ppm]	N/A	≤ 4160	N/A	< 3333
Acetone [ppm]	< 300	N/A	≤ 1000 [a]	N/A
EtOH (w/v)	< 10%	≤ 79000 ppm	< 10% [a]	7-12%
Endotoxins [EU/mL] [a]	≤ 17.5	≤ 14	< 175/*V*	< 0.125
Appearance [liquid]	Clear, colorless	Clear, colorless	Clear, colorless	Clear, colorless
Filter integrity [psi]	45	> 50	> 50	> 29
^{11}C ID [%]	≥ 99	≥ 99 [b]	≥ 99 [c]	> 99.9
^{11}C $t_{½}$ [min]	20.4±2.0	19.9-20.9 [b]	19.9-20.9 [c]	19.9-20.9
γ-spec. [keV]	511	551 [b]	486-536 [c]	511
Stability [min] [d]	60	60	100	60
Bioburden [c,e]	≤ 1 cfu/10 mL	≤ 1 cfu/10 mL	< 1 cfu/9 mL	< 10 cfu/100 mL
Sterility [a,c]	Sterile	Sterile	Sterile	Sterile

[a] After release for human use.
[b] When target gas is replaced.
[c] Periodically, every 20th batch or 6 months.
[d] Periodically, once pr. year.
[e] Not released for human use.

Table S2: Quality control equipment and parameters. Where $V_{injection}$ is the injection volume, T is the temperatures of the different equipment components (GC only), and RT is the retention times of the respective compounds in subscript.

	**AUH**	**HUH**	**OUH**	**RH**
**Dose calibrator**	CAPINTEC CRC-PC Smart Chamber	Veenstra VDC-505	Capintec CRC-15 PET	ISOMED 2010
**Endotoxin test**	Charles River PTS Endosafe	Charles River Endosafe-MCS	Charles River PTS Endosafe	Charles River Nexgen-pts
**GC**	PE, Autosystem GC, ARNEL	Shimadzu GC 2030 AF	ThermoFischer Trace 1310	Shimadzu GC 2014
Column	DB-WAX	Zebron ZB-WAX, 30 m×0.53 mm× 1.00 µm	Varian Capillary column VF-200 ms, 30 m×0.32 mm	Zebron ZB-WAX, 30 m×0.53 mm× 1.00 µm
Detector	FID	FID	FID	FID
Eluent gas	He	N_2	He	N_2
$V_{injection}$ [µL]	1	0.5	0.5	0.25
$T_{injector}$	200	150	220	230
T_{oven} [°C]	40	50	40 [a]	70
T_{FID} [°C]	200	200	270	230
Flowrate [mL/min]	7	11.2	2.7	4.7
Run time [min]	10	2.5	9.5	4
$RT_{2\text{-butanone}}$ [min]	N/A	1.5	N/A	2.4
$RT_{acetone}$ [min]	2.1	N/A	3.9	N/A
RT_{EtOH} [min]	4.0	1.8	2.4	2.6
**HPLC**	Dionex 3000 UHPLC	Shimadzu LC-20AD	Hitachi Chromaster	Dionex 3000, UHPLC
Column	Phenomenex Luna C18(2), 5 µm, 150×4.6 mm	Phenomenex Kinetex C18, 2.6 µm, 50×4.6 mm	Phenomenex Luna C18(2), 5 µm, 150×4.6 mm	Phenomenex Kinetex C18, 2.6 µm, 100 Å, 50×4.6 mm
UV detector (wavelength)	MWD-3000 (330 nm)	Shimadzu SPD-M20A (350 nm)	Hitachi 5430 (225 nm)	Dionex UltiMate 3000, DAD (370 nm)
γ-detector	Gabi Raytest	ScanSys HPLC GM	Bioscan FC-4100	ScanSys HPLC GM
Method	Isocratic	Isocratic	Isocratic	Isocratic
Run time [min]	6	7	9	3
Eluent ratio (A:B)	45:55	60:40	60:40	61:39
A	70 mM NaH_2PO_4	0.1 M NH_4HCO_2	15 mM H_3PO_4	0.1 M NH_4HCO_2
B	Acetonitrile	Acetonitrile	Acetonitrile	Acetonitrile
$V_{injection}$ [µL]	20	20	20	50
Flowrate [mL/min]	1.5	0.5	1.2	1.5
$RT_{6\text{-OH-BTA-0}}$ [min]	1.5	2.3	3.1	0.8
RT_{PiB} [min]	2.2	3.9	4.6	1.4
**pH**	pH-strips [2.0-9.0]	pH-strips [5.2-7.2]	SevenCompact pH-meter	Knick Portamess pH-meter

[a] After 5 min, the temperature is increased to 250 °C (rate: 50 °C/min) and held for 0.5 min.

S6: Synthesis Module Columns - Details and Preparation

The various synthesis modules at AUH, OUH, HUH, and RH use water and I_2 traps, as well as columns, for both transport of the gas phase and gas phase reactions. These are detailed in this section for each of the four sites.

TRACERlab FX C Pro: Water Traps and Columns at AUH

The I_2 system at AUH, for generation of [^{11}C]CH_3I, consists of two I_2 traps, a water trap, and a porapak column, whereas the system for generation of [^{11}C]CH_3OTf consists of a AgOTf column (summarized in Table S1). Whereas the I_2 is placed in a dedicated reaction vessel. An efficient AgOTf column is significant for the [^{11}C]PiB yields, and proper preparation and conditioning of the column is therefore important. After packing, the AgOTf column is conditioned for at least 1 h at 200 °C with a flow of helium. Before every synthesis, the column is conditioned for 30-60 min at 200 °C with a flow of helium.

Table S3: Packing materials and dimensions of the columns and traps for the TRACERlab FX C Pro at AUH.

**Column/Trap:**	**I_2 trap #1**				
Material:	Glass wool 5-8 µm	Ascarite II 20-30 MESH	Glass wool 5-8 µm	Empty	
Amount:	1.5 cm (Outlet)	4.0 cm	1.5 cm	5.0 cm (Inlet)	
**Column/Trap:**	**I_2 trap #2**				
Material:	Glass wool 5-8 µm	Ascarite II 20-30 MESH	Glass wool 5-8 µm	Empty	
Amount:	1.5 cm (Outlet)	4.0 cm	1.5 cm	5.0 cm (Inlet)	
**Column/Trap:**	**Water trap**				
Material:	Glass wool 5-8 µm	Sicapent with indicator	Glass wool 5-8 µm	Ascarite II 20-30 MESH	Glass wool 5-8 µm
Amount:	1.0 cm	7.5 cm	1.0 cm	7.5 cm	1.0 cm
**Column/Trap:**	**Porapak column (CH_3I trap)**				
Material:	Glass wool 5-8 µm	Porapak Q 50-80 mesh	Glass wool 5-8 µm		
Amount:	1.0 cm	4.0 cm (0.30 g)	1.0 cm		
**Column/Trap:**	**AgOTf column**				
Material:	Glass wool 5-8 µm	Carbopack™ 80-100 mesh	AgOTf (99+%)	Carbopack™ 80-100 mesh	Glass wool 5-8 µm
Amount:	1.0 cm	200 mg	200 mg	200 mg	1.0 cm

TRACERlab FX C Pro: Water Traps and Columns at OUH

At OUH, the packing of the columns and traps follow the GE user manual for the TRACElab FX C Pro equipment and is summarized in Table S2. As for the other sites, the Porapak column and AgOTf columns are conditioned when (re)packed at 200 °C/30 min (He flow: 100 mL/min) and 200 °C/60 min (He flow: 100 mL/min), respectively, as no conditioning may result in poor production yields. I_2 for the iodination reaction is placed in a dedicated reaction vessel on the TRACERlab FX C Pro equipment.

Table S4: Packing materials and dimensions of the columns and traps for the TRACERlab FX C Pro at OUH.

**Column/Trap:**	**I_2 trap #1**				
Material:	Glass wool 5-8 µm	Ascarite II 20-30 MESH	Glass wool 5-8 µm	Empty	
Amount:	1.5 cm (Outlet)	4.0 cm	1.5 cm	5.0 cm (Inlet)	
**Column/Trap:**	**I_2 trap #2**				
Material:	Glass wool 5-8 µm	Ascarite II 20-30 MESH	Glass wool 5-8 µm		
Amount:	1.5 cm	7.0 cm	1.5 cm		
**Column/Trap:**	**Water trap**				
Material:	Glass wool 5-8 µm	Sicapent with indicator	Glass wool 5-8 µm	Ascarite II 20-30 MESH	Glass wool 5-8 µm
Amount:	1.0 cm (Inlet)	3.5 cm	1.0 cm	3.5 cm	1.0 cm (outlet)
**Column/Trap:**	**Porapak column (CH_3I trap)**				
Material:	Glass wool 5-8 µm	Porapak Q 50-80 mesh	Glass wool 5-8 µm		
Amount:	0.8 cm	3.0 cm (0.30 g)	2.5 cm		
**Column/Trap:**	**AgOTf column**				
Material:	Glass wool 5-8 µm	Graphpac GC 80/100 mesh (0.6 g) + AgOTf (0.3 g)		Glass wool 5-8 µm	
Amount:	0.8 cm	3.0 cm		2.5 cm	

TracerMaker: Water Traps and Columns at HUH and RH

The columns and traps for both the I_2 and AgOTf parts on the TracerMaker system at both HUH and RH follow the manufacturers user guide and is summarized in Table S3. The I_2 used for the column should be in flakes or crystals to maximize the surface area for reaction. The I_2 used at HUH is bought from Sigma-Aldrich (326143-100G). The dry ascarite used for the various traps are either fine (20-30 mesh) or coarse (8-20 mesh). The wet ascarite is prepared by slowly mixing 50 g ascarite (8-20 mesh) with 5 mL water in a 250 mL bluecap bottle. The mixing is done by dropwise addition of water and stirring, staring with 1 mL, the procedure is repeated after >20 min until the water is used. This will prevent formation of large lumps of wet ascarite. The AgOTf column material is prepared by a mix of 1 g AgOTf and 1 g Carbopack in a vial, before loaded into the column. The column mixture is then activated by a helium flow of 50 mL/min through the column, and stepwise heating; 50 °C to 250 °C over a period of 20 min and afterwards kept at 250 °C for 30 min before cooled to RT.

Table S5: Packing materials and dimensions of the columns and traps for the TracerMaker at HUH and RH.

**Column/Trap:**	**Water trap #1**						
Material:	Glass/Quartz wool	Ascarite	Glass/Quartz wool	P_2O_5	Glass/Quartz wool		
Amount:	1.0 cm	1.5 cm	1.0	1.5 cm	1.0 cm		
**Column/Trap:**	**Water trap #2**						
Material:	Glass/Quartz wool	P_2O_5	Glass/Quartz wool	Ascarite	Glass/Quartz wool		
Amount:	1.0 cm	8.0 cm	1.0 cm	2.0 cm	1.0 cm		
**Column/Trap:**	**I_2 column**						
Material:	Glass/Quartz wool	I_2 crystals	Quartz wool				
Amount:	2.0 cm	6.4 cm	2.0 cm				
**Column/Trap:**	**I_2 trap**						
Material:	Glass/Quartz wool	Ascarite, coarse, dry	Ascarite, coarse, wet	Ascarite, fine, dry	Glass/Quartz wool	P_2O_5	Glass/Quartz wool
Amount:	1.5 cm	3.0 cm	9.0 cm	6.0 cm	1.0 cm	3.0 cm	1.5 cm
**Column/Trap:**	**AgOTf Column**						
Material:	Glass/Quartz wool	1:1 AgOTf/ carbopack	Glass/Quartz wool				
Amount:	2.0 cm	5.0 cm	2.0 cm				

ScanSys: Water Traps and Columns at RH

The columns and traps for both the I_2 and AgOTf parts on the ScanSys system at RH follow the manufacturers user guide and is summarized in Table S4. The packing adheres to the manufacturer guidelines for the ScanSys and is the same as for the TracerMaker system.

Table S6: Packing materials and dimensions of the columns and traps for the ScanSys at RH.

**Column/Trap:**	**Water trap #1**						
Material:	Glass/Quartz wool	Ascarite	Glass/Quartz wool	P_2O_5	Glass/Quartz wool		
Amount:	1.0 cm	1.5 cm	1.0	1.5 cm	1.0 cm		
**Column/Trap:**	**Water trap #2**						
Material:	Glass/Quartz wool	P_2O_5	Glass/Quartz wool	Ascarite	Glass/Quartz wool		
Amount:	1.0 cm	8.0 cm	1.0 cm	2.0 cm	1.0 cm		
**Column/Trap:**	**I_2 column**						
Material:	Glass/Quartz wool	I_2 crystals	Quartz wool				
Amount:	2.0 cm	6.4 cm	2.0 cm				
**Column/Trap:**	**I_2 trap**						
Material:	Glass/Quartz wool	Ascarite, coarse, dry	Ascarite, coarse, wet	Ascarite, fine, dry	Glass/Quartz wool	P_2O_5	Glass/Quartz wool
Amount:	1.5 cm	3.0 cm	9.0 cm	6.0 cm	1.0 cm	3.0 cm	1.5 cm
**Column/Trap:**	**AgOTf Column**						
Material:	Glass/Quartz wool	1:1 AgOTf/ carbopack	Glass/Quartz wool				
Amount:	2.0 cm	5.0 cm	2.0 cm				
